# Effectiveness of Cognitive Remediation in Subjects with Major Depressive Disorder: 3-Month Follow-Up Results of a Multicenter Randomized Controlled Study

**DOI:** 10.1192/j.eurpsy.2026.10618

**Published:** 2026-07-31

**Authors:** A. Baglioni, U. M. Battelino, L. Bertoni, S. Paolini, D. Bortoletti, R. Alberti, L. Pascariello, S. De bellis, G. Nibbio, I. Calzavara-Pinton, G. Deste, S. Barlati, A. Vita

**Affiliations:** 1 Department of Clinical and Experimental Sciences; 2 Department of Molecular and Translational Medicine, University of Brescia; 3 Department of Mental Health, ASST Valcamonica; 4 Department of Mental Health and Addiction Services, ASST Spedali Civili of Brescia, Brescia, Italy

## Abstract

**Introduction:**

Major Depressive Disorder (MDD) is among the most prevalent psychiatric conditions worldwide. Cognitive impairment is a core feature, often persisting after remission and worsening with relapses. Cognitive Remediation (CR) is a structured psychosocial intervention targeting cognition and psychosocial functioning. This study reports the 3-month follow-up (T2) results of a previously conducted RCT, which assessed outcomes at baseline (T0) and post-treatment (T1).

**Objectives:**

To evaluate the sustained effects at 3 months (T2) of computerized CR versus an active control (computer games, CG) in adults with MDD experiencing a Major Depressive Episode (MDE), focusing on cognitive performance, depressive symptoms, and functioning.

**Methods:**

This single-blind, multicenter RCT randomized patients to either CR, delivered by trained therapists, or CG. Outcomes were measured with validated instruments by blind assessors at T0, T1, and T2. Mixed models for repeated measures (MMRM) with an intent-to-treat analysis were applied. Inclusion criteria required ≥8 years of education; exclusions included neurological disorders, severe head injury, or intellectual disability. Drop-out was defined as>5 days interruption of antidepressant therapy or ≥2 consecutive missed sessions with declared discontinuation.

**Results:**

Of 142 screened patients, 101 were randomized (CR n = 52, CG n = 49), and 81 completed the study through T2 (CR n = 45, CG n = 36). At the 3-month follow-up, CR showed superior outcomes in self-reported depression (BDI, Figure 1, p = 0.003), subjective cognitive impairment (PDQ-5, p = 0.007), executive function (TMT-B, p = 0.032), and processing speed (DSST, Figure 2, p = 0.009), with significantly greater improvements compared to CG. No significant between-group differences were observed for clinician-rated depression (MADRS, p = 0.112) or verbal memory (CVLT, p = 0.083), although CR patients showed greater within-group improvements. While no statistically significant effects were found for TMT-A (p = 0.349) or Clinical Global Impression (CGI-S, p = 0.458), CR patients exhibited trends toward improved processing speed (TMT-A, p = 0.055) and a more pronounced reduction in clinical severity (CGI-S), suggesting potential non-specific benefits of the intervention.

**Image 1:**
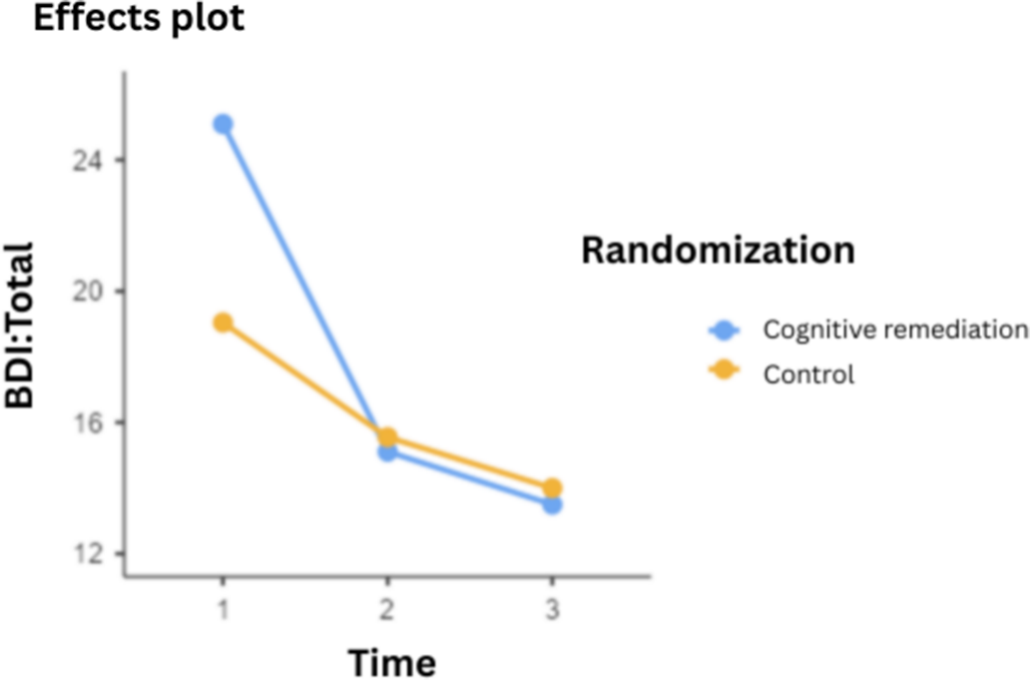
Image 1: Long description.

**Image 2:**
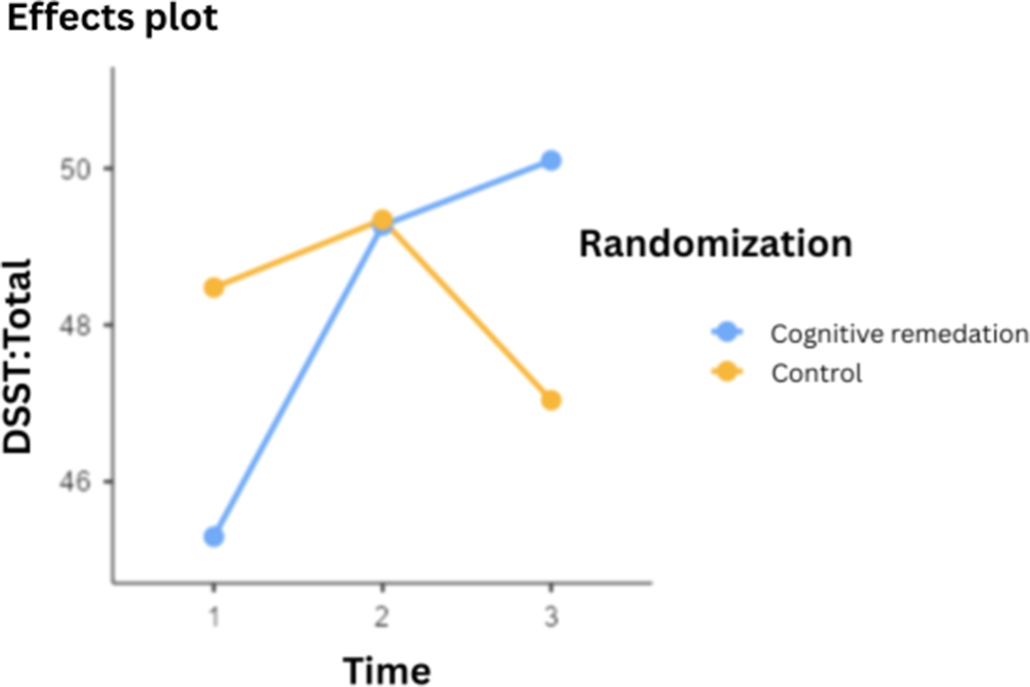
Image 2: Long description.

**Conclusions:**

This multicenter study demonstrates that CR yields sustained improvements in self-reported depression, subjective cognitive impairment, executive function, and processing speed at 3-month follow-up. Although some outcomes showed non-significant trends favoring CR, these findings support its potential as an adjunctive intervention for MDD, warranting further investigation in real-world settings.

**Disclosure of Interest:**

None Declared

## Long descriptions

### Image 1: Long description

The x-axis is labeled Time with discrete points 1, 2, and 3. The y-axis is labeled B D I dot Total with numerical increments from 12 to 24. A legend titled Randomization identifies two groups.

* Cognitive remediation (blue line): Starts at the highest point of approximately 25 at Time 1, shows a steep linear decrease to approximately 15 at Time 2, and a further slight decrease to approximately 13.5 at Time 3.

* Control (orange line): Starts at approximately 19 at Time 1, decreases to approximately 15.5 at Time 2, and ends at approximately 14 at Time 3.

The Cognitive remediation group begins with higher scores but ends with slightly lower scores than the Control group by Time 3.

Navigate back to Image 1:.

### Image 2: Long description

The x-axis is labeled Time with three discrete points marked 1, 2, and 3. The y-axis is labeled D S S T colon Total with numerical values ranging from 46 to 50. A legend to the right under the heading Randomization identifies two groups.

* Cognitive remediation (blue line): Starts at the lowest point of approximately 45.3 at Time 1, shows a steep linear increase to approximately 49.3 at Time 2, and continues with a more gradual linear increase to approximately 50.1 at Time 3.

* Control (orange line): Starts at approximately 48.5 at Time 1, increases slightly to meet the blue line at approximately 49.3 at Time 2, and then shows a sharp linear decrease to approximately 47.1 at Time 3.

Navigate back to Image 2:.

